# Social determinants of health and health-related quality of life in individuals with isolated dystonia

**DOI:** 10.3389/dyst.2025.13711

**Published:** 2025-03-12

**Authors:** Caroline Nelson, Christopher D. Stephen, Ellen B. Penney, Hang Lee, Elyse R. Park, Nutan Sharma, Marisela E. Dy-Hollins

**Affiliations:** 1 Department of Neurology, Massachusetts General Hospital, Boston, MA, United States; 2 Harvard Medical School, Boston, MA, United States

**Keywords:** dystonia, social determinants of health, quality of life, health services research, movement disorders

## Abstract

**Background and objectives:**

Dystonia is the third most common movement disorder. Motor and non-motor manifestations of dystonia may impact Health Related Quality of Life (HRQoL), with lower HRQoL scores compared to the healthy population. People with generalized dystonia report worse HRQoL scores (vs. people with focal distributions). Social determinants of health (SDOH) may play a role in HRQoL outcomes in dystonia, but scant data exists. We aimed to examine differences in HRQoL scores in people with focal vs. non-focal (e.g., segmental, multifocal, generalized) dystonia and the association with SDOH.

**Methods:**

129 participants with isolated dystonia, who were recruited through Mass General Brigham movement disorders clinic and enrolled in the Dystonia Partners Research Bank, completed a follow-up survey on SDOH and HRQoL: Quality of Life in Neurological Disorders Version 2.0 Short Form (Neuro- QoL-SF) and the EuroGroup 5-level (Euro-QoL). Linear regression analyses were performed.

**Results:**

Participants with isolated dystonia were predominantly female (72.1%), non-Hispanic white (79.8%), and highly educated (79.8%; ≥ bachelor’s degree). 71.3% of the participants had focal dystonia and 28.7% of the participants had non-focal dystonia. Participants with focal dystonia (vs. non-focal dystonia) reported older age at diagnosis (49.2 ± 11.7 vs. 40.6 ± 19.2, p = 0.004). Participants with focal dystonia (vs. non-focal dystonia) reported higher (i.e., better) overall health scores (80.4 ± 13.9 vs. 72.8 ± 13.5, p = 0.005), higher ability to participate in social activities (51.3 ± 7.7 vs. 47.2 ± 6.0, p = 0.003), lower fatigue (44.7 ± 8.4 vs. 49.8 ± 7.2, p = 0.001), and lower sleep disturbance (48.0 ± 8.2 vs. 53.0 ± 7.9, p = 0.002). Independent predictors of higher overall health ratings included focal distribution of dystonia (b = 7.5; p = 0.01), a higher level of education (b = 9.2; p = 0.04) and not having a mental health diagnosis (b = 7.5; p = 0.01).

**Conclusion:**

Participants with focal dystonia were diagnosed later and had higher (i.e., better) HRQoL measures vs. participants with non-focal dystonia. Predictors of better HRQoL were having focal dystonia and higher level of education, whereas the presence of a mental health diagnosis was associated with lower HRQoL (i.e., worse) scores. SDOH such as employment status, medical literacy, and ability to afford basic needs may influence HRQoL ratings for participants with isolated dystonia. Our findings may not be generalizable to the general population of patients with isolated dystonia. We highlight areas for further research and development.

## Introduction

Dystonia is a movement disorder characterized by sustained or intermittent muscle contractions, leading to abnormal, often repetitive movements or postures [1]. It is the third most common movement disorder [2]. Non-motor manifestations of dystonia include anxiety, depression, pain, and cognitive dysfunction [2–5]. Motor and non-motor manifestations of dystonia may impact Health Related Quality of Life (HRQoL).

HRQoL, an individual’s or a group’s perceived physical and mental health, can offer valid insights into health outcomes and indicate unmet needs to inform broader public health policy text [3, 4]. Within the context of isolated dystonia (i.e., no other motor features present), a chronic health condition that is treated symptomatically rather than curatively, HRQoL can indicate areas that current standards of care do not address.

Individuals with focal, segmental, multifocal and generalized dystonia report lower HRQoL outcome measures compared to the general population [2, 5]. In a single study, participants with generalized dystonia reported worse HRQoL scores than participants with focal distributions [6]. However, in a systematic review focused on HRQoL outcomes in dystonia participants, less than 15% of studies included participants with segmental or generalized dystonia [5].

The US Department of Health and Human Services describes Social Determinants of Health (SDOH) as the conditions in the places where people live, learn, work, and play that affect health risks and outcomes [7]. SDOH are highly relevant to neurological disorders, as neurobiological differences have been associated with SDOH, such as socioeconomic status [8]. In a study examining hippocampal brain volume in cognitively unimpaired individuals, researchers found that individuals living in areas of greater socioeconomic disadvantage (as measured through the Area Deprivation Index) were associated with lower hippocampal volume, indicating a potential aging risk marker for brain volume loss, even after adjusting for individual-level educational attainment, age, and sex [8].

There is growing evidence of associations between SDOH and various health outcomes across neurological conditions [9–13]. However, literature on SDOH in individuals with dystonia is scant. There is evidence for sex-related differences in treatment outcomes and care management for individuals with cervical dystonia, with male participants less likely to experience an objective benefit with botulinum toxin treatment, based on neurologist-rated examination, and a higher likelihood to discontinue botulinum toxin treatment compared to females [14]. Literature on ethnicity as a SDOH for individuals with dystonia primarily focuses on increased prevalence of genetically-ascribed idiopathic torsion dystonia within Ashkenazi Jewish individuals [15, 16].

Across movement disorders, there is evidence of racial health inequities, despite absent genetic justification or biological differences to account for differential health outcomes [9, 12]. Racial inequity, healthcare access, and treatment education may explain the observed differences [12]. In a review of individuals receiving deep brain stimulation (DBS) for dystonia management, Black patients were five times less likely to receive DBS compared to white patients [9, 12].

Sex may influence HRQoL outcome measures in people living with dystonia: females with cervical dystonia reported worse overall health scores on the Euro-Qol [17]; females with spasmodic dysphonia reported greater patient-perceived hoarseness [18]; and females with idiopathic blepharospasm reported significantly worse perception of general health [19].

Socioeconomic status may influence symptom impact scores of people with spasmodic dysphonia, with an association between a lower Hollingshead Four Factor Index of Socioeconomic Status (a measure of social status based on marital status, retired/employment status, educational status and occupational prestige), and worse vocal quality [20].

In this study, we aimed to examine differences in HRQoL in participants with focal dystonia vs. non-focal dystonia and assess for the role of SDOH in HRQoL outcome measures. We hypothesized that participants with focal dystonia would have better (i.e., higher) HRQoL scores compared to those with non-focal dystonia (i.e., segmental, multifocal or generalized dystonia) and that SDOH may be associated with HRQoL outcomes.

## Materials and methods

### Standard protocol approvals, registrations, and patient consents

The institutional review board at Mass General Brigham (MGB) approved this research under study Protocol# 2011P000110. Written informed consent was obtained from all participants (or guardians of participants) prior to data collection.

#### Subject population

Participants with confirmed isolated dystonia previously enrolled in the Dystonia Partners Research Bank (DPRB) were recontacted for follow-up data collection related to SDOH and HRQoL measures. Eligibility criteria for enrollment in the DPRB included a diagnosis of isolated primary dystonia disorders. Confirmation of diagnosis, including a review of medical and family history, symptom presentation, and previous medication exposure, and determination of the distribution of dystonia were provided by a neurologist trained in movement disorders (NS, MDH, EP, CS) at the time of enrollment, and at the time of follow-up data collection according to published criteria [1].

### Social determinants of health (SDOH) survey

Study data were collected and managed using Research Electronic Data Capture (REDCap) electronic data capture tools hosted by MGB Research Computing, Enterprise Research Infrastructure & Services (ERIS) group. REDCap is a secure, web-based application designed to support data capture for research studies [21].

SDOH measures were self-reported through a survey that included variables such as gender, biological sex, ethnicity, race, level of educational attainment, and medical literacy (see Supplementary Table S1).

Categorical binary demographic descriptors were created owing to limited sample size. Given Given the small number of individuals from racial (11.62%) and ethnic (8.53%) minority groups in the cohort, race and ethnicity were collapsed as non-Hispanic white vs. Other (Hispanic white, Black, American Indian, Asian, Native Hawaiian, More than one race). Similarly, as the cohort was highly educated (79.84% receiving a bachelor’s degree or graduate/professional degree), educational attainment was categorized as received less than a bachelor’s degree vs. received a bachelor’s degree or higher graduate/professional degree. Preferred language was categorized as speaking only English vs. another language (more English than another language, equally English than another language, another language more than English). Employment status was categorized as working (i.e., full-time, part-time, or full-time student) vs. not working (i.e., retired; caring for home or family, not employed not looking for paid work; unemployed, looking for work; unable to work due to illness or disability). Marital status was categorized as married vs. other (single, never been married, widowed, divorced or separated). Medical literacy was measured by asking participants to indicate their confidence filling out medical forms on a 4-point Likert-scale (all of the time, most of the time, some of the time, or none of the time). Responses were categorized as confident all of the time vs. other (most, some, or none of the time). Social needs were measured by asking participants how difficult it is to meet basic needs of living, such as paying for food, housing, medical care, and heating on a 3-point Likert scale (not hard at all, somewhat hard, and very hard). The variable was categorized into not hard at all vs. other (somewhat hard or very hard).

### Health related quality of life (HRQoL) measures

Participants completed the following HRQoL Measures: 1) Quality of Life in Neurological Disorders Version 2.0 Short Form (Neuro-QoL-SF) [22] and 2) EuroGroup 5-level (Euro-QoL) [23].

Subdomains of the Neuro-QoL are emotional behavioral dysregulation, ability to participate in social activities, fatigue, sleep disturbance, and cognitive functioning [22]. The Neuro-QoL measures the extent to which individuals reflect domain content, i.e., higher scores for negative subdomains, such as fatigue, reflect worse QoL outcomes, whereas higher scores for positive subdomains, such as the ability to participate in social activities, reflect better QoL outcomes. Neuro-QoL raw scores are calculated into respective t-scores, utilizing scale-specific conversion tables. In the healthy U.S. population, all Neuro-QoL subdomain scores are a mean of 50 and a standard deviation (SD) of 10.

The Euro-QOL measures overall health rating on a 0 to 100 sliding scale, referred to as health number [23]. Further, participants rank problem-level (i.e., no problems, slight, moderate, severe problems) related to mobility, self-care, usual daily activities, and anxiety or depression. Due to the small sample size of this study, problem severity was categorized as no problems vs. any problem (slight, moderate, or severe).

### Data availability

Anonymized data not published within this article may be made available by request from a qualified investigator.

### Statistical analysis

Data were analyzed using Stata 17.0 (College Station, TX). Due to the small sample size, distribution groups of dystonia in each type of dystonia, participants were collapsed into binary groups: 1) focal dystonia (isolated cervical dystonia, laryngeal dystonia, limb dystonia, hand dystonia, craniofacial dystonia and task specific dystonia); and 2) non-focal dystonia (multi-focal dystonia, segmental dystonia, and generalized dystonia).

The primary outcomes were HRQoL measures: emotional behavioral dysregulation, ability to participate in social activities, fatigue sleep disturbance, cognitive function, overall health number, and problems with mobility, self-care, usual activities, and anxiety/depression.

Due to the limited sample size and variability in participants’ responses, to examine the role of SDOH on HRQoL, HRQoL measures were stratified by binary groupings for employment status, medical literacy, and social needs.

Categorical variables were compared with χ^2^ or Fisher’s exact test. For continuous variables two-sample t-tests were used, and Wilcoxon rank sum test for highly skewed data.

Correction for multiple comparisons on ten HRQoL measures was conducted using Bonferroni correction and adjusted for the probability of a type I error accordingly.

Linear regression analysis was performed to investigate the impacts of covariates of HRQoL measures, namely: focal distribution (i.e., focal vs. non-focal), anxiety, depression, mental health diagnosis, sex, race (i.e., white vs. non-white), marital status (i.e., married vs. not married), preferred language (i.e., only English vs. another language), and attainment of a bachelor’s degree or higher, medical literacy, and social needs (see Supplementary Table S2). Statistical significance was set at two-tailed p < 0.05.

## Results

129 participants with isolated dystonia who were previously enrolled in the DPRB were recontacted and participated in a follow-up study on SDOH and QOL. Focal dystonia (71.3%) was more common among participants than non-focal dystonia (28.7%) (Table 1).

Participants were predominantly female (72.1%), Non-Hispanic white (79.8%) and highly educated (79.8% with a bachelor’s degree or higher). Most participants spoke only English as their preferred language (89.9%) and were married (68.2%). Mean age (SD) was 61.8 ± 13.4 years at the time of data collection (Table 2).

Most participants owned a home (77.5%), owned a car (91.5%), had high medical literacy (81.4%), and were able to meet social needs without hardship (76.7%) (Table 3). Participants were predominantly cis-gendered (98.6%) and indicated sexual orientation as straight or heterosexual (92.3%). Most participants were employed (32%) or retired (37.1%). All participants had health insurance coverage, mainly employer-sponsored insurance (40.1%) or Medicare (40.9%) (Supplementary Table S1).

Participants with focal dystonia (vs. participants with non-focal dystonia) were older at the time of assessment (63.7 ± 10.5 vs. 57.1 ± 18.0 years; p = 0.01), had a later age of symptom onset (44.3 ± 14.2 vs. 33.5 ± 22.0 years, p = 0.003), and a later age at diagnosis of dystonia (49.2 ± 11.7 vs. 40.6 ± 19.2 years, p = 0.004) (Table 2). There were no statistically significant differences in time to dystonia diagnosis (p = 0.2), sex (p = 0.99), race/ethnicity (p = 0.26), education (p = 0.99), preferred language (p = 0.52), employment status (p = 0.87), or marital status (p = 0.92) (Table 2).

SDOH characteristics (e.g., shared households, self-reported yearly income, medical literacy, social needs, car ownership, housing type) comparing participants with focal dystonia and non-focal dystonia are shown in Table 3. Participants with focal dystonia (vs. participants with non-focal dystonia) reported significantly higher median (inter-quartile range) yearly income ($145,000 [$90,000, $200,000] vs. $60,000 [$50,000, $200,000], p = 0.04). Participants with focal dystonia were more likely to report the highest level of medical literacy (i.e., confident filling out medical forms all of the time) than those with non-focal dystonia (89.1% vs. 62.2%; p = 0.001). Assessing social needs (assessed by difficulty paying for household basics: food, housing, medical care, heating, etc.), participants with focal dystonia were more likely to not have any difficulties paying compared to participants with non-focal dystonia (83.7% vs. 59.5%; p = 0.003). Participants with focal dystonia vs. participants with non-focal dystonia were more likely to own a car (95.7% vs. 81.1%; p = 0.013).

HRQoL measures in participants with focal and non-focal dystonia are shown in Table 4. On the Neuro-QoL, participants with focal dystonia vs. participants with non-focal dystonia reported higher ability to participate in social activities (51.3 ± 7.7 vs. 47.2 ± 6.0, p = 0.003), lower fatigue (44.7 ± 8.4 vs. 49.8 ± 7.2, p = 0.001), lower sleep disturbance (48.0 ± 8.2 vs. 53.0 ± 7.9, p = 0.002), and higher cognitive function (50.8 ± 8.2 vs. 47.3 ± 8.0, p = 0.02).

On the Euro-QoL, participants with focal dystonia vs. participants with non-focal dystonia reported higher overall health scores (80.4 ± 13.9 vs. 72.8 ± 13.5, p = 0.005), were less likely to report problems with mobility (29.1% vs. 51.5%, p = 0.02), and less likely to report problems performing their usual activities (29.1% vs. 54.6%, p = 0.01).

On linear regression analysis, independent predictors of higher overall health ratings on the Euro-QoL included focal distribution (b = 7.48; p = 0.008), and a higher level of education (b = 9.25; p = 0.004), and not having a mental health diagnosis (b = −7.58; p = 0.012) (Supplementary Table S2).

Table 5 examines the relationship between employment status (working vs. not working) on HRQoL measures. Individuals who were working reported less sleep disturbance than unemployed individuals (47.3 ± 9.4 vs. 51.5 ± 7.1, p = 0.005). Participants who were employed were also less likely to report problems with mobility (23.6% vs. 46.4%, p = 0.03), and performing usual activities (23.6% vs. 48.2%, p = 0.02) than participants who were unemployed.

In Table 6, HRQoL measures were compared between individuals who indicated confidence filling out medical forms all of the time and individuals who indicated they were confident filling out medical forms most, some, or none of the time. Compared to individuals who reported some level of difficulty filling out medical forms, individuals with the highest level of medical literacy had higher scores on ability to participate in social activities (50.8 ± 7.3 vs. 47.0 ± 7.5, p = 0.005), and cognitive function (50.5 ± 8.0 vs. 46.5 ± 8.6, p = 0.02). Individuals with the highest medical literacy also reported lower fatigue (45.2 ± 8.4 vs. 50.3 ± 7.2, p = 0.004) and sleep disturbance (48.7 ± 8.3 vs. 52.7 ± 8.5, p = 0.02). Individuals who were less confident filling out medical forms were more likely to report problems related to mobility (61.9% vs. 29.6%, p = 0.01), self-care (19.1% vs. 4.1%, p = 0.03), and performing usual activities (66.7% vs. 29.6%, p = 0.002), compared to those who were confident filling out medical forms all of the time (Table 6).

HRQoL outcomes were examined between individuals who experienced no hardship paying for basics such as food, housing, medical care, and heating compared to individuals who found meeting these needs somewhat or very hard (Table 7). Compared to individuals who reported some level of hardship vs. those with no difficulty scored higher on ability to participate in social activities (51.2 ± 7.3 vs. 46.5 ± 6.9, p = 0.001), cognitive function (51.2 ± 7.8 vs. 45.2 ± 8.5, p = 0.002), and overall health rating (79.9 ± 12.4 vs. 72.9 ± 18.2, p = 0.01). Individuals with no problems meeting their basic needs also had lower scores related to emotional behavioral dysregulation (45.9 ± 7.1 vs. 50.1 ± 7.7, p = 0.004), fatigue (44.6 ± 8.6 vs. 51.1 ± 5.3, p = 0.0001), and sleep disturbance (48.2 ± 8.0 vs. 53.6 ± 8.5, p = 0.001) compared to participants who reported that it was somewhat or very difficult to afford basic necessities. Individuals who reported providing for their basic needs as somewhat or very hard were more likely to report problems performing their usual activities (60.7% vs. 28.6%, p = 0.003), and self-care (17.9% vs. 3.3%), p = 0.02) (Table 7).

## Discussion

This study explored: 1) if individuals with focal dystonia had better HRQoL compared to individuals with non-focal dystonia, and 2) the role of SDOH on HRQoL measures.

Our study found that participants with focal dystonia fared better on HRQoL measures compared to participants with non-focal dystonia. Participants with focal dystonia reported lower HRQoL measures related to fatigue and sleep disturbance, higher HRQoL scores related to their ability to participate in social activities, and higher overall health ratings than participants with non-focal dystonia. Participants with focal dystonia were also less likely to report problems related to mobility and performing usual daily activities. Our findings are consistent with previous reports where individuals with focal dystonia have higher HRQoL compared to individuals with generalized dystonia [6]. Our results may indicate an extension of this trend that participants with focal dystonia report better HRQoL measures than participants with non-focal dystonia, which may be relevant for clinical treatment plans.

Independent predictors of higher overall health ratings included focal dystonia distribution, having a bachelor’s degree or higher, and not having a mental health diagnosis. These findings are consistent with a study of individuals with cervical dystonia which identified educational attainment, and clinical anxiety and depression scores as predictors for HRQoL measures, with higher education predicting better HRQoL reports and higher anxiety and depression scores predicting worse HRQoL outcomes [24].

We also explored the role of SDOH in participants with dystonia. While numerous SDOH factors were recorded, little variation was found within the participants in our study sample. However, there were differences observed in responses on SDOH from participants with focal dystonia compared to participants with non-focal dystonia. Individuals with focal dystonia reported higher annual income, were more likely to own a car, indicated no difficulty affording necessities of living, and reported higher medical literacy than individuals with non-focal dystonia.

Current literature shows that SDOH may vary across individuals with movement disorders. A previous study found that people with cerebellar ataxia, functional movement disorders, and Huntington’s disease were more likely to live in neighborhoods where residents had less than a high school diploma and an annual income of <$15,000 per year compared with individuals diagnosed with Parkinson’s disease [25]. However, this study did not include dystonia patients.

Our results contribute to the growing literature that social demographics vary across movement disorders, and potentially between subgroups of dystonia. The demographic makeup of total participants included in our study indicates a need for further research exploring SDOH in individuals across movement disorder diagnostic groups, including dystonia. The progressive nature of genetic neurodegenerative diseases such as the cerebellar ataxias and Huntington’s disease may have a profound effect on SDOH, across generations, that is not found in isolated genetic causes of dystonia. In addition, our study indicates a potential direction for SDOH research in the field of dystonia, one that includes data collection regarding medical literacy, ability to pay for basic life necessities, employment status, and the respective impacts these factors may have on HRQoL. These steps are necessary to develop interventions on mitigating disparities in individuals with dystonia [26].

The study has limitations. First, we recognize that there is selection bias. Our participant population is homogenous compared to the general population in Massachusetts, which limited our ability to evaluate all the demographic and socioeconomic factors comprehensively. The lack of diversity in a relatively homogenous participant population may be partially explained by our recruitment methods. The participants were identified through the DPRB and were recruited through the MGB outpatient movement disorder clinics. Treatments varied among the participants that included physical therapy, medications, chemodenervation with botulinum toxin, and massage that limited the ability to make meaningful comparisons. In addition, the treatments may have varied over time.

Second, the participants in our study were predominantly non-Hispanic white and highly educated and may also be explained by our recruitment process. In an analysis of 2006–2013 Medical Expenditure Panel Survey (MEPS), Black and Hispanic patients were less likely to be seen by an outpatient neurologist compared to white patients [13]. Black patients were also more likely to receive care in the emergency department and inpatient services compared to white and Hispanic patients [13]. In addition, in a recent meta-analysis associations were found with lower medical literacy and underutilization of healthcare services, and lower adherence to medication and treatment regimens for chronic conditions [27]. In a review of health literacy within neurology patients, individuals with lower health literacy were more likely to be men, African American, covered by public insurance, and earn less than $10,000 per year [10]. Thus, it is possible that those with higher health literacy are more likely to maintain long-term care for dystonia, possibly explaining the homogeneous demographics of the participants in our study. This may indicate a need for future studies to focus recruitment efforts across hospital departments, care services, and at the time of initial assessment.

We also recognize that there was low representation from sexual and gender minority groups. Only one participant identified belonging to a gender minority group. The limited self-reports of SGM may be indicative of a report bias to disclose potentially sensitive information. Further research on SGM groups within the context of neurological movement disorders is much needed, as there are no quantitative studies on the prevalence of SGM patients seen at primary movement disorder clinics [11]. Additionally, sex differences and gender affirming hormone treatments may impact the presentation of movement symptoms for SGM groups. Estrogen and testosterone are shown to modulate dopaminergic activity in the nigrostriatal pathway, which may impact symptom presentation [11, 28].

Further, the sample size and distribution of dystonia did not allow for optimal stratification of participants with different types of dystonia and association with quality of life and social determinants of health. We acknowledge that comparing participants with focal and non-focal dystonia limits our ability to extrapolate from these results. However, we collapsed the groups for higher statistical power. This also limits the generalizability of our findings. The oversimplification into binary categories may influence outcomes and interpretations. We acknowledge that certain types of focal dystonia can impact an individual’s quality of life such as writer’s cramp. Due to the low number of participants with writer’s cramp, further analysis could not be performed and is an area that could be studied in the future. In addition, the underlying etiology of dystonia (e.g., metabolic or structural causes) and/or presence of other neurodevelopmental disorders may affect quality of life measures more than other causes (e.g., primary genetic causes).

We highlight areas for future investigation that may provide meaningful insight into future investigations of SDOH for individuals with dystonia [26]. Future research should seek to increase diversity through recruitment efforts partnered with community health centers, general neurology clinics, and individuals who receive in-patient neurological services. These efforts may produce a more diverse and representative sample, in which the nuances of the role of SDOH in affecting HRQoL in dystonia patients, can be better understood. Subsequent efforts should address upstream, structural determinants of health, such as stable housing, access to reliable transportation, and social inequities such as structural racism and healthcare policies which may result in the observed social differences [29, 30].

## Supplementary Material

Supplementary Material

## Figures and Tables

**TABLE 1:** Distribution grouping of dystonia participants in SDOH HRQoL study.

Distribution	N (%)

Focal	92 (71.3)

Non-Focal	37 (28.7)

Focal	N = 92
Isolated Cervical Dystonia (Spasmodic Torticollis)	59 (64.1)
Isolated Laryngeal Dystonia (Spasmodic Dysphonia)	16 (17.4)
Isolated Limb Dystonia^a^	3 (3.3)
Isolated Hand Dystonia^b^	7 (7.6)
Task Specific Focal Dystonia^c^	4 (4.3)
Isolated Craniofacial Dystonia	3 (3.3)

Non-Focal	N = 37
Multi-Focal	14 (37.8)
Generalized	10 (27.1)
Segmental	10 (27.1)
Hemidystonia	3 (8.1)

Abbreviations: SDOH, Social Determinants of Health; HRQoL, Health Related Quality of Life.

a Dystonia affecting arm or leg.

b Writer’s cramp (n = 4) or other hand dystonia.

c Musician’s dystonia and lower extremity specific dystonia.

**TABLE 2:** Demographic characteristics of dystonia participants in SDOH HRQoL study.

	All dystonia participants N = 129	Focal distribution N = 92	Non-focal distribution N = 37	P-value

Continuous Measures	Mean (SD)/Median [Q1, Q3]	Mean (SD)/Median [Q1, Q3]	Mean (SD)/Median [Q1, Q3]	

Mean Age at Survey, years	61.8 (13.4)	63.7 (10.5)	57.1 (18.0)	0.01

Mean Age of Symptom Onset, years	41.3 (17.4)	44.3 (14.2)	33.5 (22.0)	0.003

Mean Age of Diagnosis, years	46.8 (14.7)	49.2 (11.7)	40.6 (19.2)	0.004

Median Time to Diagnosis, years	2 [0, 6]	1.5 [0, 5]	2 [1, 12]	0.2

Categorical Measures	N (%)	N (%)	N (%)	

Sex				0.99
Female	93 (72.1)	66 (71.7)	27 (73.0)	
Male	36 (27.9)	26 (28.3)	10 (27.0)	

Race/Ethnicity Collapsed^a^				0.26
Non-Hispanic White	103 (79.8)	78 (84.8)	25 (67.6)	
Other (Hispanic and/or Black, American Indian, Asian, Native Hawaiian, more than one race)	26 (20.2)	14 (15.2)	12 (32.4)	

Level of Education				0.99
Less than Bachelor’s Degree (8th grade or less, 9–11th grade, High school graduate or GED, Post high school training other than college, Associate degree, or some college)	26 (20.2)	18 (19.6)	8 (21.6)	
Bachelor’s or Graduate/Professional Degree	103 (79.8)	74 (80.4)	29 (78.4)	

Preferred Language Collapsed^b^				0.52
Only English	116 (89.9)	84 (91.3)	32 (86.5)	
Another Language (more English than another language, equally English than another language, another language more than English)	13 (10.1)	8 (8.7)	5 (13.5)	

Employment				0.87
Working	55 (47.4)	38 (46.9)	17 (48.6)	
Not Working (retired; caring for home or family, not employed not looking for paid work; unemployed, looking for work; unable to work due to illness or disability)	61 (52.6)	43 (53.1)	18 (51.4)	

Marital Status				0.92
Married	88 (68.2)	63 (68.5)	25 (67.6)	
Not Married (single, never been married, widowed, divorced or separated)	41 (31.8)	29 (31.5)	12 (32.4)	

Abbreviations: SDOH, Social Determinants of Health; HRQoL, Health Related Quality of Life; SD, Standard Deviation; Q1, First Quartile; Q3, Third Quartile

a Race/Ethnicity Collapsed: Non-Hispanic White = White, Non-Hispanic; Other = White, Hispanic; Black, Asian, American Indian, More than One Race and/or Hispanic.

b Language Collapsed: Another Language = more English than another language, equally English than another language, another language more than English.

**TABLE 3:** Social determinants of health characteristics of dystonia participants.

	All dystonia participants N = 129	Focal distribution N = 92	Non-focal distribution N = 37	P-value

Continuous Measures	Median [Q1, Q3]	Median [Q1, Q3]	Median [Q1, Q3]	

Shared Household^a^	2 [2, 3]	2 [2, 3]	2 [2, 3]	0.78

Income, $^b^	115,000.4 [70,000.0, 200,000.0]	145,000.0 [90,000.0, 200,000.0]	60,000.0 [50,000.0, 200,000.0]	0.04

Categorical Measures	N (%)	N (%)	N (%)	

Medical Literacy^c^				0.001
All of the time	105 (81.4)	82 (89.1)	23 (62.2)	
Other (Most, some, none of the time)	24 (18.6)	10 (10.9)	14 (37.8)	

Social Needs^d^				0.003
Not hard at all	99 (76.7)	77 (83.7)	22 (59.5)	
Other (Somewhat/very hard)	30 (23.3)	15 (16.3)	15 (40.5)	

Own a Car				0.013
Yes	118 (91.5)	88 (95.7)	30 (81.1)	
No	11 (8.5)	4 (4.4)	7 (18.9)	

Housing Type				—
Rent	26 (20.2)	16 (17.4)	10 (27.0)	
Own	100 (77.5)	75 (81.5)	25 (67.6)	
Other	3 (2.3)	1 (1.1)	2 (5.4)	

Abbreviations: SDOH, Social Determinants of Health; HRQoL, Health Related Quality of Life; Q1, First Quartile; Q3, Third Quartile

a Shared Household = How many individuals - adults and children - currently live in your household?

b Income = self-reported yearly income.

c Medical Literacy = How confident are you filling out medical forms.

d Social Needs = How hard is it for you to pay for the very basics like food, housing, medical care, and heating.

**TABLE 4:** HRQoL measures of dystonia participants by distribution.

	All dystonia participants N = 129	Focal distribution N = 92	Non-focal distribution N = 37	P-value

Continuous Measures	Mean (SD)	Mean (SD)	Mean (SD)	

**Neuro-QoL T scores**				
Emotional Behavioral Dysregulation	46.9 (7.5)	46.4 (7.5)	48.4 (7.2)	0.09
Ability to Participate in Social Activities	50.1 (7.5)	51.3 (7.7)	47.2 (6.0)	0.003^a^
Fatigue	46.1 (8.4)	44.7 (8.4)	49.8 (7.2)	0.001^a^
Sleep Disturbance	49.5 (8.4)	48.0 (8.2)	53.0 (7.9)	0.002^a^
Cognitive Function	49.8 (8.3)	50.8 (8.2)	47.3 (8.0)	0.02^a^

**Euro-QoL**				
Health Number	78.3 (14.2)	80.4 (13.9)	72.8 (13.5)	0.005^a^

Categorical Measures	N (%)	N (%)	N (%)	

Mobility				0.02^a^
Any problems^b^	42 (35.3)	25 (29.1)	17 (51.5)	

Self-Care				0.22
Any problems^b^	8 (6.7)	4 (4.7)	4 (12.1)	

Usual Activities				0.01^a^
Any problems^b^	43 (36.1)	25 (29.1)	18 (54.6)	

Anxiety/Depression				0.15
Any problems^b^	65 (54.6)	43 (50.0)	22 (66.7)	

a Statistically significant value, adjusted with the Bonferroni correction to account for the probability of type I error.

b Problem severity was collapsed, Any problems = slight, moderate, or severe problems combined.

**TABLE 5:** HRQoL measures of dystonia participants by employment status.

	All participants N = 121	Working N = 51	Not working N = 55	P-value

Continuous Measures	Mean (SD)	Mean (SD)	Mean (SD)	

**Neuro-QoL T scores**				
Emotional Behavioral Dysregulation	46.94 (7.5)	45.62 (7.0)	48.0 (7.8)	0.05
Ability to Participate in Social Activities	50.1 (7.5)	50.93 (6.7)	49.8 (8.2)	0.21
Fatigue	46.1 (8.4)	46.04 (8.0)	47.0 (8.4)	0.27
Sleep Disturbance	49.5 (8.42)	47.32 (9.4)	51.5 (7.1)	0.005^a^
Cognitive Function	49.8 (8.3)	50.61 (7.7)	48.7 (8.5)	0.11

**Euro-QoL**				
Health Number	78.3 (14.2)	79.4 (14.0)	78.5 (14.8)	0.37

Categorical Measures	N (%)	N (%)	N (%)	

Mobility				0.03^a^
Any problems^b^	42 (30.7)	13 (23.6)	26 (46.4)	

Self-Care				0.12
Any problems^b^	8 (5.8)	1 (1.8)	6 (10.7)	

Usual Activities				0.02^a^
Any problems^b^	43 (31.4)	13 (23.6)	27 (48.2)	

Anxiety/Depression				0.85
Any problems^b^	65 (47.5)	25 (50.0)	29 (51.8)	

a Statistically significant value, adjusted with the Bonferroni correction to account for the probability of type I error.

b Problem severity was collapsed, Any problems = slight, moderate, or severe problems combined.

**TABLE 6:** HRQoL measures of dystonia participants by medical literacy.

	All dystonia participants N = 121	All of the time N = 98	Other (most, some, none of the time) N = 23	P value

Continuous Measures	Mean (SD)	Mean (SD)	Mean (SD)	

**Neuro-QoL T scores**				
Emotional Behavioral Dysregulation	46.9 (7.5)	46.8 (7.6)	47.5 (6.8)	0.35
Ability to participate in Social Activities	50.1 (7.5)	50.8 (7.3)	47.0 (7.5)	0.005^a^
Fatigue	46.1 (8.4)	45.2 (8.4)	50.3 (7.2)	0.004^a^
Sleep Disturbance	49.5 (8.4)	48.7 (8.3)	52.7 (8.5)	0.02^a^
Cognitive Function	49.8 (8.3)	50.5 (8.03)	46.5 (8.6)	0.02^a^

**Euro-QoL**				
Health Number	78.3 (14.2)	79.3 (13.7)	73.6 (15.8)	0.05

Categorical Measures	N (%)	N (%)	N (%)	

Mobility				0.01^a^
Any problems^b^	42 (35.3)	29 (29.6)	13 (61.9)	

Self-Care				0.03^a^
Any problems^b^	8 (6.7)	4 (4.1)	4 (19.1)	

Usual Activities				0.002^a^
Any problems^b^	43 (36.1)	29 (29.6)	14 (66.7)	

Anxiety/Depression				0.48
Any problems^b^	65 (54.6)	52 (53.1)	13 (61.9)	

Medical Literacy = How confident are you filling out medical forms.

a Statistically significant value, adjusted with the Bonferroni correction to account for the probability of type I error.

b Problem severity was collapsed, Any problems = slight, moderate, or severe problems combined.

**TABLE 7:** HRQoL measures of dystonia participants by ability to meet social needs.

	All dystonia participants N = 121	Not hard at all N = 92	Other (somewhat/Very hard) N = 29	P value

Continuous Measures	Mean (SD)	Mean (SD)	Mean (SD)	

**Neuro-QoL T scores**				
Emotional Behavioral Dysregulation	46.9 (7.5)	45.93 (7.1)	50.13 (7.7)	0.004^a^
Ability to participate in Social Activities	50.1 (7.5)	51.23 (7.3)	46.51 (6.9)	0.001^a^
Fatigue	46.1 (8.4)	44.58 (8.6)	51.05 (5.3)	0.0001^a^
Sleep Disturbance	49.5 (8.4)	48.16 (8.0)	53.56 (8.5)	0.001^a^
Cognitive Function	49.8 (8.3)	51.24 (7.8)	45.18 (8.2)	0.0002^a^

**Euro-QoL**				
Health Number	78.3 (14.2)	79.92 (12.4)	72.85 (18.2)	0.01^a^

Categorical Measures	N (%)	N (%)	N (%)	

Mobility				
Any problems^b^	42 (35.3)	29 (31.9)	13 (46.4)	0.16

Self-Care				
Any problems^b^	8 (6.7)	3 (3.3)	5 (17.9)	0.02^a^

Usual Activities				
Any problems^b^	43 (36.1)	26 (28.6)	17 (60.7)	0.003^a^

Anxiety/Depression				
Any problems^b^	65 (54.6)	48 (52.8)	17 (60.7)	0.52

Social Needs = How hard is it for you to pay for the very basics like food, housing, medical care, and heating?

a Statistically significant value, adjusted with the Bonferroni correction to account for the probability of type I error.

b Problem severity was collapsed, Any problems = slight, moderate, or severe problems combined.

## Data Availability

Anonymized data not published within this article will be made available by request from any qualified investigator.
