# Supplementary material for: Social determinants of health and health-related quality of life in individuals with isolated dystonia
Source: Dystonia. Author manuscript; Available in PMC 2025 Aug 29. (PMC12393166; doi:10.3389/dyst.2025.13711)
Supplement: Supplementary Material [file NIHMS2067103-supplement-Supplementary_Material.pdf]

Supplementary Material:
Table 1. SDOH Variables Collected from Dystonia Participants in SDOH HRQoL Study

Categorical Measures	All Dystonia Participants (N%)
**Sex**	129
Female	93 (72.1)
Male	36 (27.9)
**Gender Identity**	
Cis Gendered	127 (98.6)
Additional gender category	1 (.8)
Choose not to disclose	1 (.8)
**Sexual Orientation**	
Straight or heterosexual	119 (92.3)
Lesbian, gay, or homosexual	9 (7.0)
Bisexual	1 (.8)
**Race**	
White	109 (84.5)
Black	4 (3.1)
Asian	4 (3.1)
Unspecified	5 (3.9)
Other	7 (5.4)
**Ethnicity**	
Non-Hispanic	112 (86.8)
Hispanic	11 (8.5)
**Level of Education**	
Less than 4-year College	26 (20.2)
4- year College and higher	103 (79.8)
**Maternal Level of Education**	
Less than 4-year College	92 (71.9)
4- year College and higher	36 (28.1)
**Paternal Level of Education**	
Less than 4-year College	73 (57.9)
4- year College and higher	53 (42.1)
**Language**	
Only English	116 (89.9)
More English than another language	7 (5.4)
Equally English and another language	4 (3.1)
Another language more than English	2 (1.2)
**Marital Status**	
Single	17 (13.2)
Married	88 (68.2)
Widowed	6 (4.7)
Divorced, separated	18 (14.0)
**Housing Type**	
Rent	26 (20.2)
Own	100 (77.5)
Other	3 (2.3)
**Medical Literacy**	
All of the time	105 (81.4)
Most of the time	20 (15.5)
Some of the time	3 (2.3)
None of the time	1 (.8)
**Social Needs**	

Very hard	2 (1.6)
Somewhat hard	28 (21.)
Not hard at all	99 (76.74)
**Own a Car**	
Yes	118 (91.5)
No	11 (8.5)
**Insurance Status**	
Employer-sponsored	65 (40.9)
Individual sponsored	17 (10.7)
Medicare	65 (40.9)
Medicaid	6 (3.8)
Other state or community program	2 (1.3)
Military healthcare	4 (2.5)
**Employment**	
Employed full-time	37 (31.9)
Employed part-time	17 (14.7)
Caring for home or family (not employed not looking for paid work)	4 (3.5)
Unemployed and looking for work	3 (2.6)
Unable to work due to illness or disability	11 (9.5)
Retired	43 (37.1)
Student	1 (.9)

Table 2. Multivariate Regression Model of SDOH Variables and HRQoL Health Number

Outcomes	Coefficient (b)	SE	t-value	p-value
**Euro-Qol**				
**Health Number**				
Focal Distribution	7.48	2.75	2.72	0.008
College	9.25	3.11	2.97	0.004
Mental Health	-7.58	2.98	-2.54	0.012
